# Mouse Models as Predictors of Human Responses: Evolutionary Medicine

**DOI:** 10.1007/s40139-015-0086-y

**Published:** 2015-07-08

**Authors:** Elizabeth W. Uhl, Natalie J. Warner

**Affiliations:** Department of Pathology, College of Veterinary Medicine, University of Georgia, Athens, GA 30602-7388 USA; Warner Consulting, P.O. Box 1185, Las Vegas, NM 87701 USA

**Keywords:** Evolution, Mouse models, Disease definitions

## Abstract

Mice offer a number of advantages and are extensively used to model human diseases and drug responses. Selective breeding and genetic manipulation of mice have made many different genotypes and phenotypes available for research. However, in many cases, mouse models have failed to be predictive. Important sources of the prediction problem have been the failure to consider the evolutionary basis for species differences, especially in drug metabolism, and disease definitions that do not reflect the complexity of gene expression underlying disease phenotypes. Incorporating evolutionary insights into mouse models allow for unique opportunities to characterize the effects of diet, different gene expression profiles, and microbiomics underlying human drug responses and disease phenotypes.

## Introduction

Mouse models have been increasingly used and have changed dramatically since the 1870s and 1880s when they were used by Robert Koch to develop his famous postulates for microbial pathogens [1]. Models based on clinical outcomes, such as those used by Koch, remain useful in predicting what will be infectious or toxic to humans as well as in studying the efficacy and safety of drug candidates [1]. However, the usefulness of animal models often is limited by failure to understand that many of the differences between species, and among individuals within a species, are driven by evolutionary adaptations to environment. Understanding and applying evolutionary principles to animal models can reduce this prediction problem, making the models more valuable. In addition, as our knowledge of evolution progresses, more models that contribute to our understanding of underlying disease mechanisms and of evolution itself, including human evolution, can be developed [2••].

The mouse is the most commonly used animal to model human disease and offers a number of advantages as an animal model. Mice are small, relatively inexpensive to maintain, easy to ship, have short generation times, and produce large numbers of offspring. Inbred strains are virtually genetically identical, and their environment can be controlled and manipulated. The mouse genome was sequenced just prior to the sequencing of the human genome, and there are well-established techniques for genetic manipulation in mice. Physiological and anatomical similarities between mice and humans are matched by substantial genetic homology. Mice share the majority of their genes with humans, and on average, the protein-coding regions of the human and mouse genomes are approximately 85 % identical (range ~60–99 %) (http://www.mouseencode.org/, http://www.genome.gov/10001345). Understanding the anatomy, physiology, and genetics of mice has allowed phenotypes to be manipulated through selective breeding and direct genetic manipulation, as well as by surgery or toxin exposure, to create numerous phenotypic disease models. In addition to modeling end-stage disease, mice are also increasingly being used to study the complex relationships between the individual and the environment that result in disease. However, while evolution is the basis for both the striking genetic homologies and numerous shared disease phenotypes that make studying mice and other animals so insightful for understanding human disease, it has generally not been considered in either the classification of disease or the development of animal models. Ignoring the role of evolution has led to increasing problems in the way traditional animal models are developed and utilized. A renewed emphasis on including evolutionary principles when designing experiments using mouse models will greatly reduce this prediction problem.

## Is It Safe? The Prediction Problem and Evolution

The prediction problem, which is the inability of mouse and other animal models to accurately predict human responses, is of increasing concern to scientists, regulators, and the public [3–6]. Lack of efficacy and unexpected toxicity based on animal model predictions has been cited as reasons why the current system of product discovery and development is becoming unsustainable [3–5, 7].

Rodent models, including the mouse, have correctly predicted human toxicity in only 43 % of the cases in one large study comparing human and animal drug toxicities [8]. Because of the low predictive value of rodents, the US Food and Drug Administration (FDA) requires drug testing to be done in at least two species, one of which is a non-rodent (i.e., one with a closer phylogenetic relationship to humans). Even with two species, the ability to use animal-derived data to predict human toxicity is only accurate in 71 % of cases [8]. Thus, drugs with substantial human toxicity have advanced into clinical trials and even entered the marketplace with serious consequences to patients, including death [8, 9]. Although difficult to quantify, another issue is that the development of therapeutic candidates that are toxic in animals but not humans may be prematurely discontinued.

There are a number of explanations for the failure of mouse models to predict human toxicity. The most obvious is that it is related to the inherent biochemical and physiological differences between mice and humans. In this context, it is important to realize that not only have mice and humans evolved to fit very different ecological niches, but also that the advantages of mouse models in terms of genetic homogeneity, and health status as well as the well-controlled environment and diet may limit their ability to predict effects in humans with their uncontrollable differences in age, health status, medications, genetic make-up, diet, and living conditions. Another explanation is that rare idiosyncratic toxicities may not occur in the number of animals tested, just as they may not occur in the relatively low number of subjects evaluated in human clinical trials [9].

Differences in absorption, distribution, metabolism, and excretion (ADME) of xenobiotics are a major cause of differences in the toxicity of xenobiotics between mice and humans [5]. Evolutionary adaptations to diet, in particular to the quantity and diversity of plant material consumed, have resulted in the species-specific addition and loss of genes for enzymes that also metabolize xenobiotics. Human adaptations to a plant-rich or omnivorous diet also explains why obligate carnivores, like the cat, are in general more sensitive to adverse drug reactions and thus make poorer models for predicting human responses than mice or even dogs, in which domestication has resulted in some genetic adaptations to tolerate plant material in the diet [10]. Although mice, like humans, consume an omnivorous diet, there are differences in the human and mouse genes coding for enzymes that metabolize xenobiotics, including the cytochrome P450 system of enzymes, which metabolize 75 % of drugs [11]. This knowledge, along with the use of pharmacokinetics in safety pharmacology and toxicology testing of drug candidates, has improved the use of mouse models for toxicity testing. Based on improved understanding of both the inter- and intra-species differences in enzymes that metabolize xenobiotics, mouse models can be more specifically targeted to predict outcomes of human exposure, potentially even idiosyncratic ones. In addition, pharmacokinetics allows exposure in animal models to be based on plasma, serum, or even tissue concentrations of the xenobiotic (and its metabolites). Future advances include “humanizing” mouse metabolism for specific or classes of xenobiotics [12].

Evolutionary adaptations to diet impact more than just the enzymes involved in xenobiotic metabolism, as diet-related changes in gene expression and the intestinal microbiome are a major source of species differences [13]. Mice offer practical ways to model these adaptations and have been used to study the effects of different human and primate diets on gene expression in both the liver and brain [13]. In one study, groups of mice were fed one of four diets: commercial mouse pellets; a chimpanzee diet consisting of vegetables, fruit, and yogurt; cooked food from a human cafeteria; and McDonald’s fast food [13]. The usefulness of mouse models in assessing dietary effects on gene expression was revealed in the observation that 10 % of the liver genes with different expression in humans and chimpanzees had altered expression in the mice fed human versus chimpanzee diets *for only two weeks* [13]. In addition, although the two human diets did not result in different gene expression in the liver, the human fast food diet was the only one that produced detectable effects on gene expression in the brain [13]. These findings raise the possibility of using mouse models to study the effects of different human diets on human disease as well as drug metabolism, efficacy, and toxicity.

Another important way adaptations to diet can affect how drugs are metabolized is through the intestinal microbiome. It has long been recognized that the microbiota can greatly impact the efficacy of therapeutic compounds [14]. The ways in which digestive tract bacteria within their hosts affect toxicant and drug metabolism are complex and occur through a wide variety of mechanisms including activation, detoxification, direct binding, altered gene expression and kinetics, production of pathway intermediates, enterohepatic cycling, and stimulation of immune responses [15•]. A ‘gut-based pharmacology’ is currently being revealed that has important implications for how drugs are administered and how they act on various body systems [16]. Because of the widespread effects on both drug toxicities and efficacies, it has recently been proposed that components of the microbiome should be considered “druggable” targets [14].

While manipulating the microbiome offers potential breakthroughs in terms of therapeutic interventions, models are needed to sort out its complex effects and identify potential targets. Mouse models have much to offer in this regard [17•]. Studies in which microbiota from an individual human are transplanted into mice have yielded promising research insights. For example, transplantation of fecal microbiota from adult human twins discordant for obesity into germ-free mice has shown that the obese phenotype can be prevented through co-housing with the lean twin’s microbiota [18••]. These kinds of studies indicate the extent to which an ‘omics’ base approach using mice can be used to make better predictive models of human drug reactions and disease. The power of this approach is that the reactions of specific groups or even individual humans can be modeled, especially when used together with the types of genetic manipulation possible in mice. However, before the full technological potential for the development of more predictive mouse models can be realized, another important source of the prediction problem needs to be addressed: the inadequacy of the current disease definitions.

## Does It Work? Enhancing the Predictive Value of Mouse Models by Incorporating Evolution into Disease Definitions

The disease definitions currently in use are an important factor underlying the failure of mouse models to predict drug efficacy and safety. Recent developments in genome-based technologies have shown that existing definitions of disease based on phenotype are overly broad, encompassing subsets of patients with different underlying mechanisms in a single entity, such as hypertension, obesity, diabetes, asthma, and Alzheimer’s Disease. Animal models based on the current disease definitions have focused on artificially creating some, usually the primary, characteristic(s) of the disease being targeted. Even when the overall or average response to treatment in the human population has been sufficiently well predicted in these models to result in a drug that could be marketed, subsets of patients who do not respond or who develop toxicities have remained. Incorporation of evolutionary principles into disease definitions and the development of animal, particularly mouse, models are already occurring and will improve the predictive value of mouse models and allow individualized, personalized, or precision medicine.

Previous paradigm shifts in defining disease have been preceded and followed by significant advances in technology and medicine. For example, the re-classification of disease as infectious or not in the late 1800s to early 1900s was preceded by the development of tissue staining and microbial culture techniques, which directly led to Koch’s postulates. These new technologies also led to the germ theory of disease and the subsequent development of sterile technique and antimicrobials. Today it is the rapid development of genome-based technologies that is driving medicine toward new characterizations of disease [19]. Evolutionary principles, which incorporate understanding of the differences among species as well as among individuals within a species, provide the logical basis for using the data from genome-based technologies to understand and classify disease. However, until recently medicine has been missing an evolutionary perspective. The consequence is that animal models of human disease have been based almost entirely upon presumed similarities, usually artificially created, to human disease. Differences from the human disease phenotype, which exist in almost every animal model, have been ignored, glossed over or used to put down a rival model in the competition for funding. As a result, the genetic and molecular complexity underlying many disease phenotypes has been underestimated until revealed by failure of various animal models to predict responses in human patients [3]. The situation is now beginning to change. Genetic and genomic testing has revealed that many disease phenotypes represent the final expression produced by multiple mechanisms. Classifications for mouse models of disease based on causative mechanism rather than clinical phenotype are being developed characterized and used (http://mouse.ncifcrf.gov and http://jaxmice.jax.org/query/f?p=205:1:0). Thus, an evolutionary perspective is increasingly critical for the effective development of disease models as emphasized by the finding that the same gene defect can produce very different phenotypes depending upon species, individual, or even tissue. This finding also indicates that the context in which a mutation is expressed will be critical to the rational discovery and development of new approaches for treating or correcting gene defects. For example, mutations in the dystrophin gene induce muscle atrophy in humans and dogs and muscle hypertrophy in domestic cats but are comparatively benign in mice [20, 21]. Compensatory mechanisms and rapid muscle regeneration both appear to be involved in ameliorating the loss of dystrophin gene function in mice, although the phenotype can be manipulated to better model the human disease by mutating the genes involved in these compensations [21]. As the differences in response to loss of a functional dystrophin gene indicate, defects in single genes known to cause diseases in humans may have very different effects in other species. Another example is Cystic fibrosis transmembrane conductance regulator (CFTR) gene-deficient mice, which developed gastrointestinal obstruction rather than the pulmonary lesions characteristic of cystic fibrosis in humans [22, 23]. Important insights into pathogenic mechanisms can be gained by studying the *differences* among phenotypes for various animal species and humans and this can yield new therapeutic targets. Evolution, as the source of these often subtle but critical differences, is the context in which they can best be understood and utilized.

## Enhancing the Value of Mouse Models: Gaining Insights from Other Species and Modeling Human Evolution

The genetic complexity that underlies human disease phenotypes make animal models even more important to understanding mechanisms of disease. In particular, mice are especially important models because so much of their gene expression has been characterized and can be easily manipulated with relative confidence in the outcome. However, the value of mice as models can be enhanced by consideration of the mechanisms behind the disease phenotypes observed in other species. For example, mutations in the dystrophin gene in cats are characterized by muscle hypertrophy, not muscle wasting, as is the case in humans and dogs; thus, the cat has not been considered as good an animal model for the human disease as the dog, even though the same gene is mutated in all three species [24]. Because reversing or slowing the devastating muscular atrophy is a therapeutic goal for Duchene’s muscular dystrophy in human patients, the hypertrophied phenotype of dystrophin-deficient cats is a warning that some mechanisms for inducing muscle hypertrophy are unlikely to be curative and could possibly even make the disease worse [20]. Given that inhibition of myostatin is a potent inducer of muscular hypertrophy and thus has been considered as a potential therapeutic approach for dystrophin deficiency-related muscular dystrophies in humans, manipulating mice to model the cat phenotype, in particular to evaluate the role of myostatin inhibition and other inducers of muscular hypertrophy in the context of dystrophin deficiency, could potentially be informative in terms of identifying better therapeutic approaches.

An exciting new application for mouse models is using them to model aspects of human evolution. Genomic information is available from a wide variety of nonhuman primates and human populations, including individuals who lived hundreds or thousands of years ago, and genotype mapping has identified numerous genes associated with specific traits or disease susceptibilities. However, confirming the association of genotypic changes with specific phenotypes is difficult, especially as humans cannot be selectively bred. Therefore, mouse models have been increasingly used to provide insights into the evolution of a variety of features including regulatory elements, synaptic densities, brain size, and speech. They can even be used to test genotype–phenotype hypotheses related to human evolution, as the correspondence of specific phenotypes to the fixed genetic changes in the human lineage can be compared by engineering mice to express an exon, gene, or genomic region from either a modern human gene or its ancestral orthologue and then analyzing the resulting phenotypes [2••]. The phenotypes can be characterized in functional assays adapted for a variety of readouts depending upon the genetic target [2••]. In addition, animals that have been ‘humanized’ for specific genes, genomic regions, or cell types can also provide insights into human evolution. For example, mice that have been engrafted with human glial cell progenitors develop astroglia that morphologically resemble those of humans and also have enhanced long-term potentiation and learning behaviors in comparison to mice engrafted with mouse glial precursors [25]. This divergence between the two lines of engineered mice shows that real genetic differences between human and mouse glial cells contribute to the species-specific functional capabilities.

## Conclusions

Recent breakthroughs in technology have allowed the mouse and human genomes to be sequenced, and partial or full gene expression profiles in healthy and diseased individuals are increasingly being characterized. It has been found that genomes among species are much more similar than originally thought, which provides a genetic as well as anatomic and physiological basis for using animals to model human diseases and predict human responses to drugs and other therapies. However, the gene expression profiles of disease phenotypes and drug responses are complex, varying not only from species to species but also from individual to individual. This variation has resulted in the ‘prediction problem,’ which is the failure of animal studies to suitably predict human responses. Important sources of the prediction problem have been the failure to consider the evolutionary basis for species differences, especially in drug metabolism, and disease definitions that do not reflect the complexity of gene expression that underlie various disease phenotypes. Incorporating evolutionary insights into the design and development of mouse models allows for unique opportunities to characterize the effects of diet, different gene expression profiles, and microbiomics underlying human drug responses and disease phenotypes.
